# A Two-Week Insulin Infusion in Intrauterine Growth Restricted Fetal Sheep at 75% Gestation Increases Skeletal Myoblast Replication but Did Not Restore Muscle Mass or Increase Fiber Number

**DOI:** 10.3389/fendo.2021.785242

**Published:** 2021-11-30

**Authors:** Eileen I. Chang, Byron Hetrick, Stephanie R. Wesolowski, Carrie E. McCurdy, Paul J. Rozance, Laura D. Brown

**Affiliations:** ^1^ Perinatal Research Center, Department of Pediatrics, Section of Neonatology, University of Colorado School of Medicine, Aurora, CO, United States; ^2^ Department of Human Physiology, University of Oregon, Eugene, OR, United States

**Keywords:** IUGR fetus, skeletal muscle, insulin, myoblast, myogenesis, sheep

## Abstract

Intrauterine growth restricted (IUGR) fetuses are born with lower skeletal muscle mass, fewer proliferating myoblasts, and fewer myofibers compared to normally growing fetuses. Plasma concentrations of insulin, a myogenic growth factor, are lower in IUGR fetuses. We hypothesized that a two-week insulin infusion at 75% gestation would increase myoblast proliferation and fiber number in IUGR fetal sheep. Catheterized control fetuses received saline (CON-S, n=6), and the IUGR fetuses received either saline (IUGR-S, n=7) or insulin (IUGR-I, 0.014 ± 0.001 units/kg/hr, n=11) for 14 days. Fetal arterial blood gases and plasma amino acid levels were measured. Fetal skeletal muscles (biceps femoris, BF; and flexor digitorum superficialis, FDS) and pancreases were collected at necropsy (126 ± 2 dGA) for immunochemistry analysis, real-time qPCR, or flow cytometry. Insulin concentrations in IUGR-I and IUGR-S were lower *vs*. CON-S (*P ≤* 0.05, group). Fetal arterial P_a_O_2_, O_2_ content, and glucose concentrations were lower in IUGR-I *vs*. CON-S (*P ≤* 0.01) throughout the infusion period. IGF-1 concentrations tended to be higher in IUGR-I *vs*. IUGR-S (*P*=0.06), but both were lower *vs*. CON-S (*P ≤* 0.0001, group). More myoblasts were in S/G_2_ cell cycle stage in IUGR-I *vs*. both IUGR-S and CON-S (145% and 113%, respectively, *P ≤* 0.01). IUGR-I FDS muscle weighed 40% less and had 40% lower fiber number *vs*. CON-S (*P ≤* 0.05) but were not different from IUGR-S. Myonuclear number per fiber and the mRNA expression levels of muscle regulatory factors were not different between groups. While the pancreatic β-cell mass was lower in both IUGR-I and IUGR-S compared to CON-S, the IUGR groups were not different from each other indicating that feedback inhibition by endogenous insulin did not reduce β-cell mass. A two-week insulin infusion at 75% gestation promoted myoblast proliferation in the IUGR fetus but did not increase fiber or myonuclear number. Myoblasts in the IUGR fetus retain the capacity to proliferate in response to mitogenic stimuli, but intrinsic defects in the fetal myoblast by 75% gestation may limit the capacity to restore fiber number.

## Introduction

Intrauterine growth restriction (IUGR) due to placental insufficiency (PI) is a common condition during pregnancy that lowers the supply of both oxygen and nutrients to the developing fetus (1, 2). Fetuses exposed to IUGR are born with smaller skeletal muscle mass compared to normally growing counterparts (3, 4). The consequences of less lean muscle mass not only affect fetal weight, but persist beyond the perinatal period into adulthood (5, 6), resulting in lasting adverse metabolic health conditions (7–10). Epidemiological studies have linked low birth weight and decreased muscle mass to the development of the metabolic syndrome and type 2 diabetes (11–13), insulin resistance (14–16), and increased risk for adverse cardiovascular events later in life (17).

PI-IUGR fetuses in an experimental fetal sheep model have lower skeletal muscle mass and myofiber number compared to normally growing fetuses (18). These fetuses also have reduced rates of myogenesis, or the capacity for the myoblast to proliferate and fuse into multinucleated myofibers by 90% of gestation (18). In addition, plasma concentrations of insulin and insulin growth factor-1 (IGF-1), both myogenic growth factors, are lower in IUGR fetuses (19). Insulin plays an important role in fetal growth and development; however, the exact role of insulin in regulating fetal skeletal muscle growth is still being elucidated.

A one-week insulin infusion directly into normally growing fetal sheep resulted in increased skeletal myoblast proliferation (20). Additionally, when we exposed IUGR fetal skeletal myocytes to insulin for five days *in vitro*, we found an increase in myoblast proliferation in a dose dependent manner (21). This suggests that supplementing insulin to IUGR fetuses might stimulate skeletal myoblast proliferation, thereby increasing myofiber number and overall skeletal muscle mass. Previous *in vivo* and *in vitro* studies with supplementary insulin were conducted on fetal sheep or skeletal myocytes harvested at 90% gestation, thereby missing the peak period for myoblast proliferation and fiber formation which begins as early as 60% gestation (22). In this study, we tested the hypothesis that a two-week insulin infusion would increase skeletal myoblast proliferation and myofiber number in IUGR fetal sheep *in vivo* beginning at approximately 75% gestation (~110 day gestation, dGA; term is 147 dGA). Given the important role that the insulin-producing pancreatic islet and β-cells play in the development of the metabolic syndrome and diabetes, we also sought to ensure that the insulin infusion did not downregulate fetal pancreatic β-cell mass.

## Materials and Methods

### Ethics Approval

**Figure 1:**
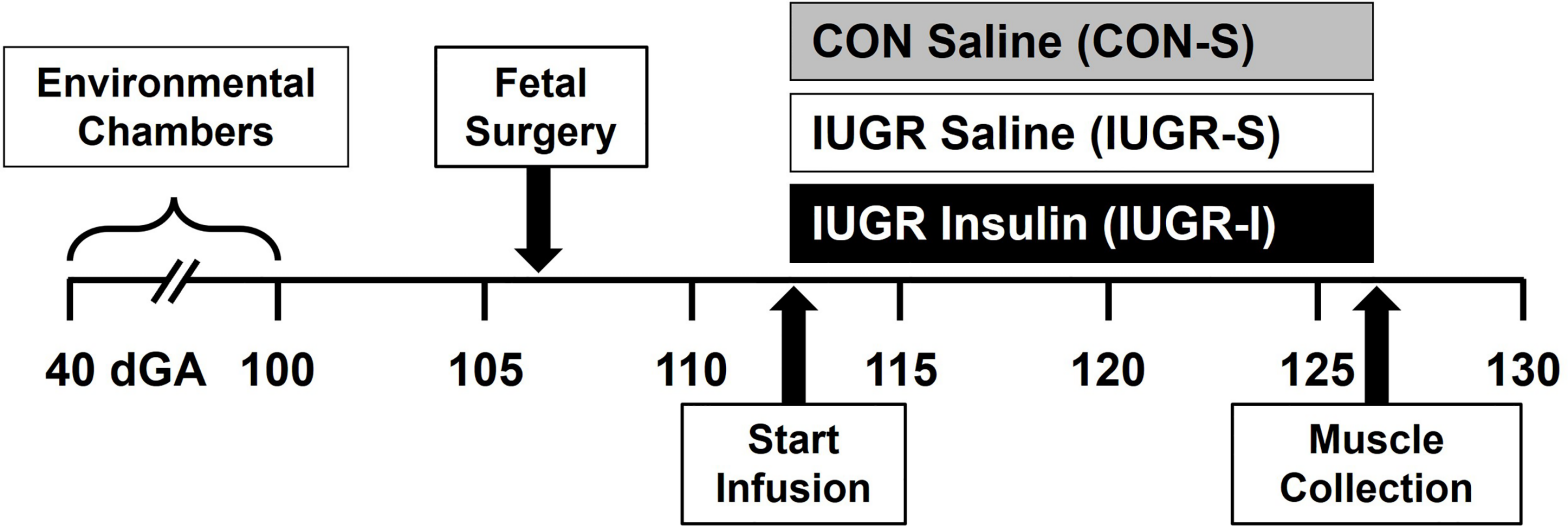
Experimental design. Pregnant ewes were randomly assigned and housed in an environmental chamber from 41 ± 2 to 99 ± 1 days gestation (dGA) with either elevated temperatures (40°C for 12 hrs; 35°C for 12 hrs; 35-40% humidity) to produce placental insufficiency and IUGR, or with ambient temperature (21°C for 24 h; 35-40% humidity) as control (CON). After surgical procedure (106 ± 1 dGA) and recovery, CON fetuses received saline (CON-S, n=6, grey) while IUGR fetuses received either saline (IUGR-S, n=7, white) or insulin infusion (IUGR-I, n=11, black) for 14 days starting on 112 ± 1 dGA. Muscles were collected at necropsy on 126 ± 2 dGA.

All animal procedures were in compliance with the guidelines of the US Department of Agriculture, the National Institutes of Health, and the Association for Assessment and Accreditation of Laboratory Animal Care International. The animal care and protocols were approved by the University of Colorado Institutional Animal Care and Use Committee [#334]. Animal studies were performed at the University of Colorado Perinatal Research Center. The manuscript is in compliance with the ARRIVE guidelines for reported animal research (23). The overall experimental design is presented in **Figure 1**.

### Animal Model and Surgical Procedures

Columbia–Rambouillet mixed-breed ewes (Nebeker Ranch, Lancaster, CA, USA) carrying singleton pregnancies and first parity, confirmed by ultrasound, were randomly assigned to produce PI-IUGR or served as control (CON). Ewes assigned to the IUGR group were housed in an environmental chamber with elevated ambient temperatures (40°C for 12 hrs; 35°C for 12 hrs; 35-40% humidity), and ewes assigned to the CON group were housed in an environmental chamber with normal ambient temperatures (21°C for 24 hrs; 35-40% humidity) from 41 ± 2 to 99 ± 1 dGA. After environmental treatment and for the remainder of the studies, ewes were housed in normal ambient temperatures and humidity in metabolic carts. Ewes were given *ad libitum* access to water, and efforts were made to match maternal feed intake between sheep in CON and IUGR groups, and all intakes were well above national standards of intake for livestock set by the National Research Council.

Ewes were fasted (24 hrs) and thirsted (12 hrs) prior to surgery. At 106 ± 1 dGA, surgery was performed using aseptic techniques to place maternal and fetal intravascular catheters as previously described (18, 20, 24). Briefly, anesthesia and antibiotic (0.2 mg/kg of diazepam, 20 mg/kg of ketamine, I.V.; 600,000 U of Penicillin G procaine, I.M.) were administered to the ewe for induction and intubation, and the ewe was maintained on 2-4% isoflurane with supplemental oxygen for the remainder of the surgical procedures. Maternal heart rate and respiration were continuously monitored while under anesthesia. The fetal hindlimb was delivered through a maternal laparotomy and hysterotomy and hindlimb lengths were measured. The fetal femoral artery from the same hindlimb was catheterized with placement of the tip in the external iliac artery, and the other hindlimb was not operated. With the fetal neck exposed, two catheters were placed alongside one another into the jugular vein. Before the uterus was sutured closed, ampicillin (500 mg) was injected into the amniotic fluid. Maternal femoral artery and vein were also catheterized after the maternal *linea alba* and skin were closed. Prior to maternal midline skin closure, local analgesic was applied to the incision (3 mL, 5 mg/mL Marcaine, 0.5% bupivacaine hydrochloride). All fetal and maternal catheters were routed subcutaneously to the left flank of the ewe and gathered into a pouch affixed to the skin. Ewes received at least six days of postoperative care before experimentation, with an initial two days of analgesic treatments and close monitoring (2.2 mg/kg of Banamine, *I.M.*, *bid*), probios (10 g of Probios, *P.O.*, *bid*).

### Experimental Design and Fetal Tissue Collection

A two-week intravenous infusion started on 112 ± 1 dGA, when CON fetuses received saline (CON-S, n=6; 6 female), while IUGR fetuses were randomly assigned to receive either saline (IUGR-S, n=7; 6 female, 1 male) or insulin (IUGR-I, n=11; 5 female, 6 male). Insulin infusion dose (0.015 units/kg/hr) and rate (0.1 to 0.4 ml/hr) was calculated from an estimated fetal weight, which was based on the hindlimb length obtained at the time of surgery. The saline infusion rate was matched to the approximate insulin infusion rate at 0.4 ml/hr. Fetal arterial blood (1 mL) was sampled at baseline (day 0) and daily thereafter to monitor blood gases (ABL 800 Flex PLUS Blood Gas Analyzer, Radiometer America Inc., Copenhagen, Denmark) and plasma glucose and lactate concentrations (YSI 2900 Biochemistry Analyzer, YSI Inc., Yellow Springs, OH, USA) throughout the infusion period. If fetal plasma glucose concentrations fell by 25% in an IUGR-I fetus, a dextrose (20%) infusion was supplemented to maintain euglycemia. Fetal arterial plasma was collected on days 0, 1, 4, 8, 11, and 14 to measure insulin, IGF-1, and cortisol concentrations by ELISA as previously described (21). To standardize the analysis for better comparisons, all blood gas and plasma hormone measurements were averaged in the following range of days: 0 (baseline), 1, 2-4, 5-8, 9-11, and 12-14 days. Plasma norepinephrine concentrations were measured on days 0, 8, and 14 using HPLC as previously described (25). Plasma amino acid (AA) concentrations were measured on days 0, 8, and 14 using a Dionex TM ICS 5000+ high pressure ion chromatograph with a Pickering PCX Pinnacle 120-4 channel variable wavelength detector (Thermo Electron North America, LLC) using methods previously described (26).

At 126 ± 2 dGA, the ewes received intravenous anesthesia (0.2 mg/kg of diazepam; 20 mg/kg of ketamine) and the uninstrumented fetal hindlimb was exposed through maternal laparotomy and hysterotomy (CON-S, n=6, 6 female; IUGR-S, n=7, 6 female, 1 male; IUGR-I, n=8, 3 female, 5 male). The infusions continued while the fetal biceps femoris (BF) muscle biopsy was collected for flow cytometry, gene expression, and immunohistochemistry analyses. The ewe and the fetus were then euthanized with a fatal dose of intravenous pentobarbital sodium (ewe: 85 mg/kg; fetus: 250 mg/kg; Fatal Plus; Bortech Parmaceuticals, Dearborn, MI, USA). The fetus, pancreas, skeletal muscle [flexor digitorum superficialis (FDS)], and other organs were weighed, measured, and stored at -80°C. FDS for immunohistochemistry was cut at mid-belly, placed on corkboard, thinly coated with OCT compound, and immediately frozen in liquid nitrogen-cooled 2-methylbutane for 1 min and stored at -80°C.

### Primary Skeletal Myocyte Isolation for Flow Cytometry

BF muscle (CON-S, n=4, 4 female; IUGR-S, n=6, 5 female, 1 male; IUGR-I, n=7, 2 female, 5 male) was immediately submerged in chilled Ham’s F-12 Nutrient Mix (Thermo Fisher Scientific, Waltham, MA, USA) and then washed with Hanks’ Balanced Salt solution (HBSS, MilliporeSigma, St. Louis, MO, USA). The connective tissue was carefully removed and muscle was digested in 1% collagenase in HBSS with gentle mincing, then incubated for 30 min at 37°C. Bovine serum albumin (0.5% BSA in HBSS) was added to the mixture in 1:1 volume ratio. The entire solution was filtered through a 70 μm cell strainer, then centrifuged at 270 x g for 10 min at 25°C. The cell pellet was washed twice in 1X phosphate-buffered saline (PBS). An aliquot of the myoblasts was stained with Live/Dead Fixable Yellow (Thermo Fisher) according to manufacturer’s instructions. All cells were then fixed in 2% paraformaldehyde for 30 min, then washed with 1X PBS and stored at 4°C in 1X PBS until flow cytometry analysis.

For cell cycle analysis, paraformaldehyde fixed myoblasts were washed in ice cold 70% ethanol and stored overnight at 4°C. Myoblasts were then washed with 1X PBS, incubation buffer (1% BSA in 1X PBS), and stained with 1 μg of CD56 primary antibody conjugated to PE/Cy5 (BioLegend, San Diego, CA, USA, Cat# 304607; RRID : AB_314449) in 0.1 mL incubation buffer for 30 min at 25°C. The purpose of staining with CD56 primary antibody was to identify myoblasts in the flow cytometer. Myoblasts were then washed with incubation buffer and stained with 3 μM of DAPI in incubation buffer (0.5 mL). All wash steps were carried out by centrifugation at 500 x g for 5 min at 25°C followed by decanting the supernatant. Data was acquired using a Gallios flow cytometer and Kaluza software (Beckman Coulter, Indianapolis, IN, USA). Gates were determined by comparing unstained and singly stained samples. In the cell cycle analysis based on DAPI staining, we only selected cells that were identified as both Live/Dead Yellow negative and CD56 positive.

### Immunohistochemistry

#### Fetal Skeletal Muscle Analysis

Total myofiber number was measured using cryopreserved FDS muscle (CON-S, n=6, 6 female; IUGR-S, n=7, 6 female, 1 male; IUGR-I, n=8, 3 female, 5 male) as previously described (18). Briefly, a full thickness section from the mid-portion of the FDS muscle was obtained and cryosectioned (5 μm). The tissues were stained with primary antibodies: anti-laminin rabbit polyclonal IgG (1:500; Sigma-Aldrich, Cat# L9393; RRID : AB_477163), anti-dystrophin mouse monoclonal IgG2b (1:250; Developmental Studies Hybridoma Bank; cat.# MANDRA1(7A10); RRID: AB_2618143); and secondary antibodies: Alexa Fluor 488 goat anti-rabbit IgG (1:200; Thermo Fisher Scientific, Cat# A-11008; RRID : AB_143165), Cy2 donkey anti-mouse IgG (1:250; Jackson ImmunoResearch Labs, Cat# 715-225-150; RRID : AB_2340826).

Myonuclear number was measured as previously described (18). Briefly, cryopreserved FDS muscle (CON-S, n=6, 6 female; IUGR-S, n=6, 5 female, 1 male; IUGR-I, n=6, 2 female, 4 male) was cryosectioned (5 μm). The tissues were stained with primary antibodies: anti-laminin rabbit polyclonal IgG (1:500; Sigma-Aldrich, Cat# L9393; RRID : AB_477163); and secondary antibodies: Alexa Fluor 488 goat anti-rabbit IgG (1:200; Thermo Fisher Scientific, Cat# A-11008; RRID : AB_143165), and counterstained with 4′,6-Diamidino-2-phenylindole dihydrochloride (DAPI) (1:1000; Sigma-Aldrich; Cat# D8417).

Whole muscle sections were imaged (IX-83 microscope; *vs*120 Virtual Slide Scanning system; Olympus, Center Valley, PA). The total myofiber number and fiber area were quantified using custom applications in Visiopharm image analysis software (Visiopharm, Hoersholm, Denmark) from the full thickness cross-section of the FDS muscle. Myofibers were identified, counted, and then converted to area (μm^2^) per fiber. Myonuclear number was quantified with 3-5 images at 20X magnification, with at least a sum of 1000 fibers counted per fetus. Any DAPI^+^ nuclei that were >50% inside the fibers (identified by laminin^+^ staining) were quantified and counted as myonuclei, and the data are expressed as number of myonuclei per myofiber.

#### Fetal Pancreas Analysis

Cryopreserved fetal pancreas (CON-S, n=5, 5 female; IUGR-S, n=7, 6 female, 1 male; IUGR-I, n=8, 3 female, 5 male) were sectioned and stained for insulin and glucagon-positive areas and vascularity as previously described (27). The tissues were stained with primary antibodies: anti-porcine insulin guinea pig polyclonal IgG (1:250, Bio-Rad, Cat# 5330-0104G; RRID : AB_1605150); anti-glucagon mouse monoclonal IgG (1:500, Sigma-Aldrich, Cat# G2654; RRID : AB_259852); and for endothelial cells – Griffonia simplicifolia lectin 1 (GSL1) isolectin B4 (15 μg/mL; Vector Laboratories, Cat# FL-1201; RRID : AB_2314663). The secondary antibodies that were used: Alexa Fluor 594 goat anti-mouse IgG (1:250; Thermo Fisher Scientific, Cat# A-11005; RRID : AB_2534073); and Alexa Flour 680 donkey anti-guinea pig IgG (1:500; Jackson ImmunoResearch Labs, Cat# 706-625-148; RRID : AB_2340478).

Images from each fetal pancreas were acquired for total pancreatic area, hormone-positive areas (4 sections), and vascularity (2 sections). The morphometric analysis was performed using custom applications in Visiopharm. The percent area of the pancreas positive for insulin or glucagon was calculated by dividing the respective hormone positive area by total pancreatic area. The β-cell and α-cell mass were calculated by multiplying the pancreas weight by the percent total insulin-positive (β-cell) and glucagon-positive (α-cell) areas, respectively. For each type of analysis, all the sections were averaged per animal prior to statistical analysis.

### mRNA Analysis of Fetal Skeletal Muscle and Pancreas

Total RNA was extracted from 300 mg of BF muscle (CON-S, n=6, 6 female; IUGR-S, n=7, 6 female, 1 male; IUGR-I, n=8, 3 female, 5 male), and 100 mg of pancreas (CON-S, n=6, 6 female; IUGR-S, n=7, 6 female, 1 male; IUGR-I, n=8, 3 female, 5 male), using 1 mL of TRIzol LS Reagent (Invitrogen). Tissues were homogenized, mixed with chloroform (200 μL/mL of homogenate), and centrifuged at 12,000 x g for 15 min at 4°C. The total RNA was isolated and purified from the aqueous phase using RNeasy Mini Kit (Qiagen) per the company’s manual, and the concentration was determined with NanoDrop 1000 Spectrophotometer (ThermoScientific). RNA (2 μg) was reverse transcribed using Superscript III and Oligo dT 18–20 (Invitrogen) at 50°C for 1 hr. Real-time qPCR (Lightcycler 480 II; Roche Life Science) on a 1:10 dilution was performed in triplicate using standard curves for relative quantification between groups as previously described (Benjamin et al., 2017). Primers were developed and validated for all real-time PCR assays shown in **Table 1**. If the complete gene sequence was not available in sheep (*Ovis aries*) at the time of primer design, then cow (*Bos taurus*) and human (*Homo sapiens*) primer sequences were also screened for homology. The identification of a single gene product and optimal efficiency of the melting curve were evaluated beforehand with all new primers designed. Gene expression was normalized to the average of three reference genes (*H3-3A*, *HMBS*, and *RHOA*) for fetal skeletal muscle, and four reference genes (*MLF*, *RPL32*, *RPL37A*, and *S15*) for fetal pancreas. mRNA expression levels of reference genes did not vary by treatment; therefore, all experimental genes are expressed as a ratio with the equal-weighted average of the reference genes.

**Table 1:** Primers used for real-time qPCR assays for fetal skeletal muscle and pancreas.

Symbol	Gene Name	Forward Primer (5’-3’)	Reverse Primer (5’-3’)	Accession #	Amplicon length (bp)	*Source*
**Skeletal Muscle**						
*PAX7*	Paired Box 7	AGC TAC CGG ACT CCA CCT A	CCC TGG TGC ATG GTG GAC	XM_616352.6	99	*Bos taurus*
*MYOD1*	Myogenic Differentiation 1	GCA ATC CGC TAT ATC GAA GG	GTA GTA AGC GCG GTC GTA GC	NM_002478.4	236	*Homo Sapiens*
*MYF6*	Myogenic Factor 6 (MRF4)	TTC AGC TAC AGA CCC AAG CAG GAA	TTG TCC CTC CTT CCT TGG CAG TTA	NM_181811.1	126	*Bos Taurus*
*MYF5*	Myogenic Factor 5	AAG GTG GAG ATC CTC AGG AA	ATT CAG GCA TGC CAT CAG AGC AAC	NM_174116.1	150	*Bos taurus*
*MYOG*	Myogenin	GCG CAC TGG AGT TTG GCC	ACT GTG ATG CTG TCC ACG AT	NM_001111325.1	105	*Bos Taurus*
*DES*	Desmin	GGA CCT GCT CAA TGT CAA GAT	AAG GTC TGG ATA GGG AGG TT	XM_004004952.1	101	*Ovis aries*
*MSTN*	Myostatin	ACG ATG TCC AGA GAG ATG ACA GCA	ATC AGA CTC CGT GGG CAT GGT AAT	NM_001009428.1	97	*Ovis aries*
*FST*	Follistatin	TGC ACT CCT CAA GGC CAG ATG TAA	ATT AGT CTG GTC CAC CAC GCA TGT	NM_175801.2	126	*Bos taurus*
*CDKN1A*	Cyclin Dependent Kinase Inhibitor 1A (P21)	GAG GAC CAC TTG GAC CTG T	TCT GCG TTT GGA GTG GTA GA	NM_001098958.1	146	*Bos taurus*
*CCND1*	Cyclin D1	ACTACCTGGACCGCTTCCT	TTGGAGAGGAAGTGCTCGAT	NM_053056.2	253	*Homo Sapiens*
*CCNE2*	Cyclin E2	TGATGGTGCTTGCAGTGAAGAGGA	GACCGTTACAGGACAAAGTTCCCA	NM_001015665.1	87	*Bos Taurus*
*CCNA2*	Cyclin A2	CCTGCAAACTGCAAAGTTGAA	GGTGAAGGTCCAGGAGACA	NM_001237.2	216	*Homo Sapiens*
*CCNB1*	Cyclin B1	TGGGTCGTGAAGTCACTGGAAACA	CAGCATCTTCTTGGGCACACAGTT	NM_001045872.1	154	*Bos Taurus*
*CDK1*	Cyclin-Dependent Kinase 1	ACACATGAGGTAGTGACACTCTGG	ATGTCCACTGGAGTTGAGTAGCGA	NM_001142508.1	79	*Ovis Aries*
*CDK2*	Cyclin Dependent Kinase 2	GGTGTACCCAGTACTGCCA	CGCAGAGGCATCCATGAATT	BT020790.1	158	*Bos taurus*
*CDK4*	Cyclin Dependent Kinase 4	ATTTCCTTCATGCCAACTGCA	CCAACACTCCACATGTCCAC	NM_000075.2	216	*Homo Sapiens*
*CDK6*	Cyclin Dependent Kinase 6	GCATCGTGATCTAAAACCACA	GAGTCCAATCACGTCCAAGA	NM_001259.5	285	*Homo Sapiens*
**Pancreas**						
*INS*	Insulin	TCA GCA AAC AGG TCC TCG CAA G	GGG CCA GGT CTA GTT ACA GTA G	BC142034.1	355	*Bos taurus*
**Reference Genes**						
*H3-3A*	H3.3 Histone A (Previous HGNC Symbol: H3F3A)	CAGAGTGGCCGCAAATCG	CAGGCCTGTAACGATGAGGTT	XM_004013633.3	118	*Ovis aries*
*HMBS*	Hydroxymethylbilane Synthase	CTGCTGGAGAAGCTGGAGAC	CGGGTACCCACTCGAATCAC	NM_001046207.1	193	*Bos Taurus*
*RHOA*	Ras Homolog Family Member A	TCTTCGAAACGACGAGCACA	AGCTCTCGTGGCCATTTCAA	NM_001161875.1	172	*Ovis aries*
*MLF2*	Myeloid Leukemia Factor 2	CTCAGCATCACAGATGGCAA	CATGTCGTTCATCATCCCAA	XM_012175508.2	135	*Ovis aries*
*RPL32*	Ribosomal Protein L32	AATCAAGCGGAACTGGCG	GGCATTGGGATTGGTGATT	XM_004018540.3	292	*Ovis aries*
*RPL37A*	Ribosomal Protein L37a	ACC AAG AAG GTC GGA ATC GT	GGC ACC ACC AGC TAC TGT TT	XM_027965159	192	*Ovis aries*
*S15*	Ribosomal Protein S15	ATC ATT CTG CCC GAG ATG GTG	CGG GCC GGC CAT GCT TTA CG	NM_001018.2	146	*Homo Sapiens*

### Statistical Analysis

One-way ANOVA or Kruskal-Wallis and Fisher’s least significant difference (LSD) *post hoc* tests were used for direct comparisons between 3 groups: CON-S, IUGR-S, and IUGR-I (Prism GraphPad Software, La Jolla, CA, USA, Version 9.2.0). For outcomes with repeated measurements *in vivo*, two-way ANOVA with Mixed Procedure was used to determine the effect of treatment (CON-S, IUGR-S, IUGR-I), time (day of infusion), and their interaction (SAS Institute Inc., Cary, NC, USA, Version 9.4). Fisher’s LSD *post hoc* test was used to determine differences between groups and time (from baseline). Significance was determined at *P ≤* 0.05; exact *P*-values are given for values of 0.1 or less. Data are displayed as mean ± standard error of the mean (SEM). We were unable to assess the effect of sex in this study due to random yet unequal distribution of sex among groups. Data for norepinephrine were log-transformed for analysis because they were not normally distributed. The differences in animal numbers per group for some analyses are due to fetal demise from surgical procedures or during the infusion period, catheter failure, and/or the availability of collected tissue for various downstream analysis.

## Results

### Fetal Growth and Physiological Parameters

At the conclusion of the 14-day infusion period (**Table 2**), IUGR-S and IUGR-I fetal body weights were 36% and 30% less, fetal crown-to-rump lengths were 13% and 12% shorter, and fetal hindlimb lengths were 18% and 14% shorter, respectively, when compared to CON-S. IUGR-S and IUGR-I individual hindlimb muscle weights were lower compared to CON-S. IUGR-S and IUGR-I fetal heart weights were 28% and 22% less, the kidneys were 29% and 22% less, and the brains were 12% and 15% less, respectively, when compared to CON-S. After normalizing to body weight, only the ratio of brain:body weight of IUGR-S fetuses was greater compared to CON-S; however, no statistical differences were found between IUGR-I and CON-S. In addition, no differences were found among the groups after normalizing the heart and kidneys to fetal body weight. The IUGR-I placentome weight was 47% lower and the number of placentomes were 24% fewer compared to CON-S. However, the ratio of fetal to placental weight was 37% higher in IUGR-I compared to CON-S.

**Table 2:** Fetal weight and length measurements at the end of the infusion period.

	CON-S	IUGR-S	IUGR-I	*P*-Value
**Fetal Parameters**				
Gestational age (d)	125 ± 1	126 ± 1	127 ± 1	0.244
Total number (n)	6	7	8	
Male (males/total)	0/6	1/7	5/8	
Fetal weight (g)	2451.1 ± 211.1^A^	1572.5 ± 195.5^B^	1718.4 ± 182.9^B^	0.016
Crown-rump length (cm)	44.5 ± 1.3^A^	38.8 ± 1.2^B^	39.3 ± 1.1^B^	0.009
Hindlimb length (cm)	31.7 ± 1.1^A^	26.0 ± 1.0^B^	27.3 ± 1.0^B^	0.004
Fetal brain:body weight ratio	20.01 ± 2.21^A^	28.58 ± 2.05^B^	24.41 ± 1.92 ^A,B^	0.036
**Organ Weight (g)**				
Pancreas	2.52 ± 0.23^A^	1.97 ± 0.22^A,B^	1.79 ± 0.20^B^	0.078
Liver	83.0 ± 10.4	60.5 ± 10.4	63.2 ± 9.0	0.264
Lungs	93.6 ± 9.6^A^	61.4 ± 8.9^B^	65.2 ± 8.4^B^	0.051
Heart	20.4 ± 1.5^A^	14.6 ± 1.4^B^	15.9 ± 1.3^B^	0.033
Kidneys	15.8 ± 1.2^A^	11.2 ± 1.1^B^	12.4 ± 1.1^B^	0.032
Adrenal	0.30 ± 0.04	0.29 ± 0.04	0.38 ± 0.03	0.150
Spleen	7.78 ± 1.33	5.30 ± 1.15	5.11 ± 1.23	0.289
Brain	47.4 ± 2.0^A^	41.9 ± 1.8^B^	40.2 ± 1.7^B^	0.038
Uteroplacental weight (g)	1248 ± 172.2	982.7 ± 114.3	901.2 ± 100.1	0.171
Placentome weight (g)	320.8 ± 42.5^A^	209.0 ± 39.3^A,B^	168.6 ± 36.8^B^	0.042
Placentome number (n)	88 ± 4^A^	80 ± 3^A^	67 ± 3^B^	0.001
Fetal:placental weight ratio	8.57 ± 1.21^A^	7.76 ± 0.60^A^	11.73 ± 1.23^B^	0.033
**Hindlimb Muscle Weights**				
Gastrocnemius (g)	5.95 ± 0.58^A^	3.75 ± 0.54^B^	3.89 ± 0.50^B^	0.021
per hindlimb length (g/cm)	0.187 ± 0.015^A^	0.141 ± 0.014^B^	0.140 ± 0.013^B^	0.063
Tibialis anterior (g)	2.70 ± 0.30^A^	1.76 ± 0.27^B^	1.71 ± 0.26^B^	0.042
per hindlimb length (g/cm)	0.084 ± 0.008	0.066 ± 0.007	0.061 ± 0.007	0.113
Flexor digitorum superficialis (g)	1.88 ± 0.23^A^	1.26 ± 0.22^A,B^	1.20 ± 0.20^B^	0.088
per hindlimb length (g/cm)	0.039 ± 0.007	0.047 ± 0.007	0.043 ± 0.006	0.721
Extensor digitorum longus (g)	0.97 ± 0.17^A^	0.41 ± 0.16^B^	0.40 ± 0.15^B^	0.043
per hindlimb length (g/cm)	0.030 ± 0.005^A^	0.015 ± 0.005^A,B^	0.014 ± 0.005^B^	0.081
Soleus (g)	0.40 ± 0.26	0.17 ± 0.28	0.58 ± 0.28	0.607
per hindlimb length (g/cm)	0.013 ± 0.009	0.007 ± 0.010	0.021 ± 0.010	0.629

All values are means ± SEM. Gestational age refers to age at the end of study. One-way ANOVA, and Fisher’s LSD post hoc test to determine differences among groups (different letters are statistically significantly different from each other, P ≤ 0.05).

**Figure 2:**
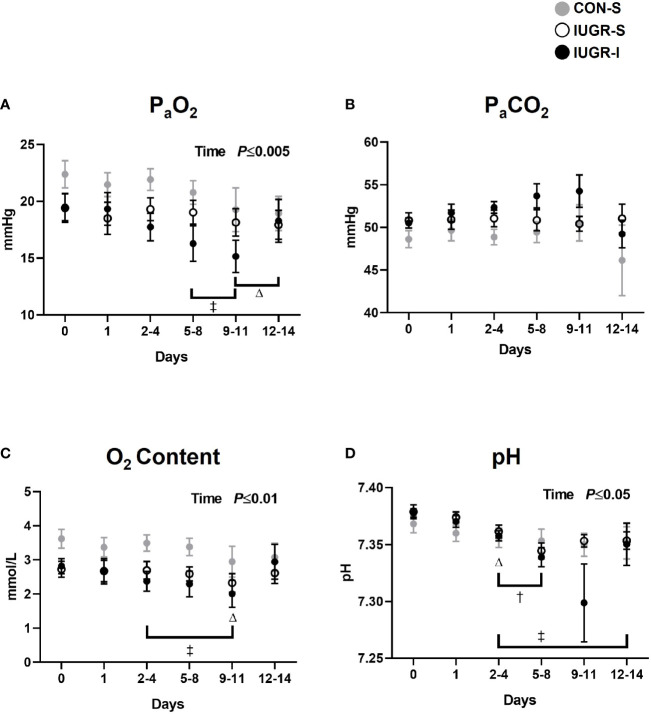
Fetal arterial blood gases. Fetal arterial **(A)** P_a_O_2_, **(B)** P_a_CO_2_, **(C)** O_2_ content, and **(D)** pH during insulin or saline infusion period. CON-S (grey circle, n=6), IUGR-S (white circle, n=7), and IUGR-I (black circle, n=11). Values are mean ± SEM. Statistical significance was designated at *P ≤* 0.05 by two-way ANOVA to determine the effects of treatment (CON-S, IUGR-S, IUGR-I), time (day of infusion), and interaction. Fisher’s least significant difference *post hoc* test was indicated by the following: Δ, CON-S *vs*. baseline; †, IUGR-S *vs*. baseline; and ‡, IUGR-I *vs*. baseline.

Fetal arterial blood gas values were measured throughout the 14-day infusion period (**Figure 2**). Compared to baseline values, IUGR-I fetal arterial blood gases showed 17-22% lower levels of arterial partial pressure of oxygen (P_a_O_2_) from days 5-8 through 9-11; 16-29% lower O_2_ content from days 2-4 through 9-11; and 0.2-1% lower pH from days 2-4 through 12-14. IUGR-S fetal pH level from days 2-4 through 5-8 was lower by 0.5% compared to baseline. Compared to baseline values, CON-S fetuses had 14-15% lower P_a_O_2_ from days 9-11 through 12-14, 19% lower O_2_ content at days 9-11, and 0.2% lower pH at days 2-4. An overall treatment effect was not detected among the three groups.

**Figure 3:**
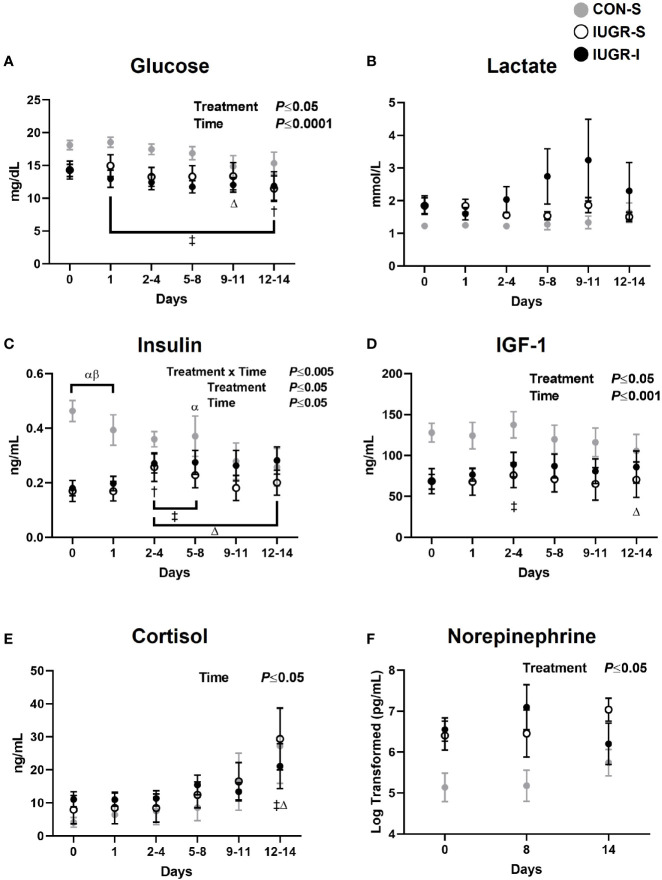
Fetal arterial plasma hormone values. Fetal arterial plasma **(A)** glucose, **(B)** lactate, **(C)** insulin, **(D)** IGF-1, **(E)** cortisol, and **(F)** norepinephrine values during insulin or saline infusions. CON-S (grey circle, n=6), IUGR-S (white circle, n=7), and IUGR-I (black circle, n=11). Values are mean ± SEM. Statistical significance was designated at *P ≤* 0.05 by two-way ANOVA to determine the effects of treatment (CON-S, IUGR-S, IUGR-I), time (day of infusion), and interaction. Fisher’s least significant difference *post hoc* test was indicated by the following: α, IUGR-S *vs*. CON-S; β, IUGR-I *vs*. CON-S; Δ, CON-S *vs*. baseline; †, IUGR-S *vs*. baseline; and ‡, IUGR-I *vs*. baseline. Data for norepinephrine were log-transformed for analysis.

For fetal hormone concentrations (**Figure 3**), plasma insulin concentrations in IUGR-S and IUGR-I fetuses at baseline were 63% and 61% lower, respectively, than in CON-S. IUGR-I fetal insulin concentrations increased by 51-53% on days 2-4 through 5-8 compared to baseline. In addition, we observed a 20-45% decrease in insulin concentrations in CON-S from day 3 to the end of the experiment compared to baseline values. The overall mean IGF-1 concentrations in IUGR-S and IUGR-I were 43% and 35% lower, respectively, compared to the CON-S (IUGR-S, 68.47 ± 13.16 ng/mL; IUGR-I, 79.02 ± 10.55 ng/mL; CON-S, 121.05 ± 14.22 ng/mL). However, IUGR-I had a 31% increase in plasma IGF-1 concentrations on days 3-4 compared to baseline. CON-S had 18% lower IGF-1 concentrations on the last day of the experiment compared to its own baseline values. Both IUGR-I and CON-S had increased plasma cortisol levels on the last day of infusion compared to their own baselines. The overall mean norepinephrine concentrations were 5-fold and 12-fold higher in IUGR-S and IUGR-I, respectively, compared to the CON-S (IUGR-S, 1241 ± 1908 pg/mL; IUGR-I, 3024 ± 1568 pg/mL; CON-S, 259 ± 2062 pg/mL).

For fetal substrate concentrations (**Figure 3**), the overall mean plasma glucose concentrations in IUGR-I fetuses were 28% lower when compared to CON-S (IUGR-I, 12.28 ± 1.00 mg/dL; CON-S, 17.06 ± 1.36 mg/dL). IUGR-I plasma glucose concentration decreased by 18% from baseline. Additionally, the glucose concentrations in CON-S and IUGR-S fetuses were also lower from baseline by 18% on days 9-11 and by 20% on days 12-14. Lactate concentrations were not significantly different among groups or over the infusion period.

**Figure 4:**
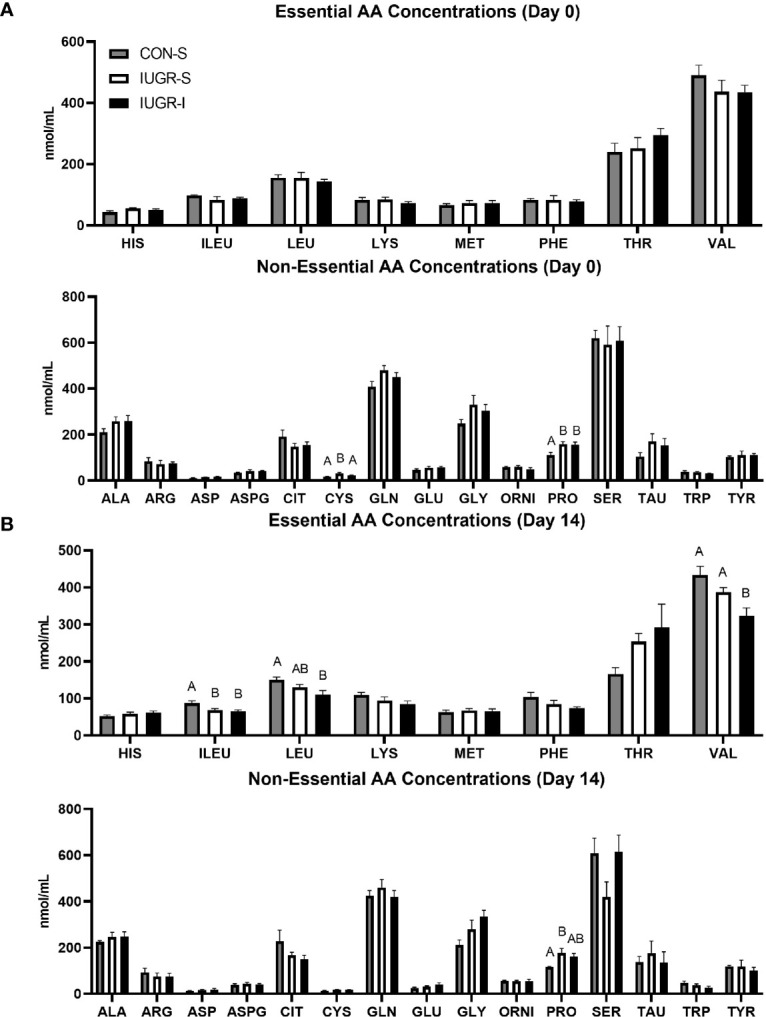
Fetal arterial plasma amino acids (AA) concentrations before infusion and on the final day of infusion. Fetal essential and non-essential AA concentrations on **(A)** day 0 and **(B)** day 14 of the infusion period. Day 1: CON-S (grey bar, n=6), IUGR-S (white bar, n=7), and IUGR-I (black bar, n=11). Day 14: CON-S (grey bar, n=4), IUGR-S (white bar, n=5), and IUGR-I (black bar, n=5). Values are mean ± SEM. Statistical significance was designated at *P ≤* 0.05 by one-way ANOVA, and Fisher’s least significant difference *post hoc* test to determine differences among groups (different letters are statistically significantly different from each other, *P ≤* 0.05).

Fetal plasma AA concentrations were measured on days 0 (baseline), 8, and 14 (final day of infusion). Only results from days 0 and 14 are shown in **Figure 4** as there were minimal differences in concentrations at the day 8 timepoint Fetal plasma AA concentrations at baseline are shown in **Figure 4A**. There were no differences in the essential AAs at baseline among groups. However, of the non-essential AAs, proline was 43% and 42% higher in IUGR-S and IUGR-I fetuses, respectively, compared to CON-S. In addition, cysteine was 75% higher in IUGR-I when compared to CON-S. Fetal plasma AA concentrations on the final day of the infusion are shown in **Figure 4B**. The concentrations of the branched chain AA (BCAA; isoleucine, leucine, and valine) were 25%, 27%, and 26% lower in IUGR-I, respectively, when compared to the CON-S. Compared to its baseline values, the IUGR-I plasma concentrations of isoleucine decreased by 25%, leucine decreased by 23%, and valine decreased by 26%. Fetal plasma alanine concentrations were not different between the groups at baseline or on the final day of the infusion period. IUGR-S fetal plasma serine concentrations decreased 29% by day 14.

### Maternal Physiological Parameters

Maternal physiological measurements are shown in **Table 3**. Although pregnant ewes were randomly assigned to a treatment group, maternal weight in IUGR-I prior to environmental chamber treatment was 14% and 15% lower compared to CON-S and IUGR-S, respectively. In addition, IUGR-I maternal weight at surgery was 17% and 13% lower compared to CON-S and IUGR-S, respectively, which may explain why maternal intake was lower. Maternal biochemical values for glucose, lactate and blood gas measurements did not differ among groups.

**Table 3:** Maternal parameters.

	CON-S (n = 6)	IUGR-S (n = 7)	IUGR-I (n = 8)	*P*-Value
**Pretreatment Measurements**				
Entry chamber weight (lbs)	121.8 ± 6.0^A^	124.1 ± 5.0^A^	105.3 ± 4.6^B^	0.029
Surgery weight (lbs)	139.3 ± 6.6^A^	133.3 ± 3.7^A^	115.9 ± 4.1^B^	0.006
**Chamber Period**				
Feed intake (lbs/day)	4.62 ± 0.19^A^	3.86 ± 0.18^B^	3.35 ± 0.17^C^	<0.0001
Water intake (L/day)	5.69 ± 0.83^A^	8.30 ± 0.77^B^	5.14 ± 0.72^A^	0.019
**Infusion Period**				
Feed intake (lbs/day)	4.74 ± 0.31^A^	3.84 ± 0.29^B^	3.68 ± 0.27^B^	0.050
Water intake (L/day)	7.27 ± 0.55	6.89 ± 0.51	6.30 ± 0.48	0.413
**Post-treatment Measurements**				
Glucose (mmol/L)	61.83 ± 1.07^A,B^	57.05 ± 2.63^A^	63.74 ± 1.48^B^	0.094
Lactate (mmol/L)	0.90 ± 0.20	0.76 ± 0.10	0.66 ± 0.10	0.529
pH	7.43 ± 0.04	7.44 ± 0.01	7.45 ± 0.01	0.819
P_a_CO_2_ (mm Hg)	34.58 ± 5.17	34.52 ± 0.92	32.62 ± 0.96	0.834
P_a_O_2_ (mm Hg)	88.25 ± 0.84	90.96 ± 1.36	90.08 ± 1.65	0.428
Hct (%)	34.65 ± 0.55^A^	30.60 ± 0.89^B^	32.22 ± 1.57^A,B^	0.096
Hemoglobin (mg/dL)	6.98 ± 0.13	6.16 ± 0.19	6.48 ± 0.33	0.113
O_2_ Content (mmol/L)	6.35 ± 0.10	5.66 ± 0.19	6.00 ± 0.30	0.160
O_2_ Saturation (%)	93.73 ± 1.05	95.10 ± 0.69	95.88 ± 0.36	0.151

All values are means ± SEM. One-way ANOVA, and Fisher’s LSD post hoc test to determine differences among groups (different letters are statistically significantly different from each other, P ≤ 0.05).

### Myoblast Cell Cycle Stage Analysis by Flow Cytometry

**Figure 5:**
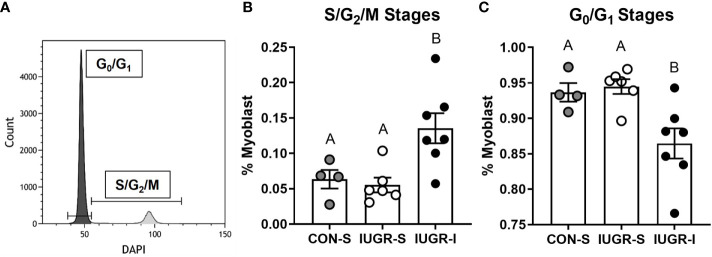
Flow cytometry analysis of cell cycle stages on fetal myoblasts. Fetal myoblasts dissociated from BF muscle were identified by CD56 marker, then stained with DAPI to identify the cell cycle stage of the myoblasts. **(A)** Representative plot to identify cell cycle stages of myoblasts using DAPI staining. **(B)** Myoblasts in the S/G_2_/M stages were 113% and 145% higher in IUGR-I *vs*. CON-S and IUGR-S, respectively. **(C)** Myoblasts in the G_0_/G_1_ stages were 7.7% and 8.5% lower in IUGR-I *vs*. CON-S and IUGR-S, respectively. CON-S (grey circle, n=4), IUGR-S (white circle, n=6), and IUGR-I (black circle, n=7). Statistical significance was designated by different alphabetical letters at *P ≤* 0.05 by one-way ANOVA and Fisher’s least significant difference *post hoc* test.

BF muscle from IUGR-I fetuses had 113% and 145% more myoblasts in the S/G_2_/M cell cycle stage when compared to CON-S and IUGR-S, respectively (**Figure 5**). In addition, the number of myoblasts in the G_0_/G_1_ cell cycle stage was lower in IUGR-I by 8% compared to CON-S and 9% compared to IUGR-S.

### Total Myofiber Number and Size, and Myonuclear Number Per Fiber

**Figure 6:**
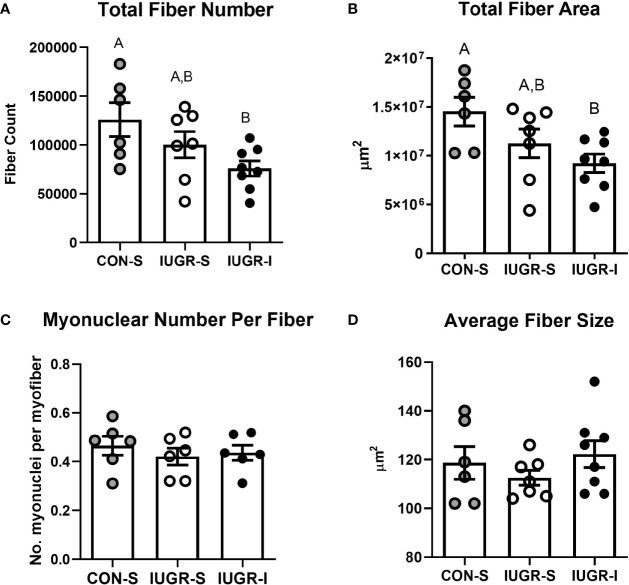
IUGR-I fetuses have lower fiber number and fiber area in the flexor digitorum superficialis (FDS) muscle compared to CON-S fetuses. **(A)** Total fiber number, **(B)** total fiber area, **(C)** myonuclear number per fiber, and **(D)** average fiber size. The IUGR-I fetuses have 40% lower total fiber number when compared to CON-S (P=0.040, one-way ANOVA). In addition, the IUGR-I fetuses have 36% less total fiber area when compared to CON-S (P=0.031, one-way ANOVA). However, the overall size of individual fibers and the number of myonuclei per fiber were not different among the groups. CON-S (grey circle, n=6), IUGR-S (white circle, n=6-7), and IUGR-I (black circle, n=6-8). Values are mean ± SEM. Statistical significance was designated by different alphabetical letters at *P ≤* 0.05 by one-way ANOVA and Fisher’s least significant difference *post hoc* test.

Total myofiber number in the FDS muscle was 40% lower in IUGR-I fetuses when compared to CON-S (**Figure 6**). In addition, the entire cross-sectional fiber area of IUGR-I fetal FDS muscle was 36% smaller when compared to CON-S. The individual fiber size, calculated by dividing the total fiber area by total myofiber number, was not different among the groups, nor were there differences among groups for myonuclear number (**Figure 6**).

### Skeletal Muscle mRNA Analysis

Normalized fetal skeletal mRNA expression of cyclin E2 (*CCNE2*) was 76% higher in IUGR-I *vs*. IUGR-S (**Table 4**). Cyclin dependent kinase 4 (*CDK4*) gene expression tended to be 19% higher in IUGR-I *vs*. IUGR-S. Cyclin D1 (*CCND1*) mRNA expression was 61% higher in IUGR-S *vs*. CON-S. The mRNA expression levels of muscle regulatory factors were not different among groups.

**Table 4:** Skeletal muscle, biceps femoris, mRNA expression (fold change from CON-S).

Genes	CON-S (n=6)	IUGR-S (n=7)	IUGR-I (n=8)	*P*-Value
**Cell Cycle Regulators**				
*CDKN1A*	1.00 ± 0.21	1.41 ± 0.23	2.45 ± 1.10	0.455
*CCND1*	1.00 ± 0.07^A^	1.61 ± 0.25^B^	1.40 ± 0.17^A,B^	0.044
*CCNE2*	1.00 ± 0.09^A,B^	0.82 ± 0.05^A^	1.44 ± 0.27^B^	0.025
*CCNA2*	1.00 ± 0.14	1.03 ± 0.13	1.05 ± 0.11	0.966
*CCNB1*	1.00 ± 0.07	0.87 ± 0.12	1.06 ± 0.14	0.504
*CDK1*	1.00 ± 0.10	0.93 ± 0.20	1.28 ± 0.26	0.461
*CDK2*	1.00 ± 0.12	1.13 ± 0.10	1.17 ± 0.05	0.404
*CDK4*	1.00 ± 0.07	0.99 ± 0.06	1.18 ± 0.06	0.066
*CDK6*	1.00 ± 0.17	1.68 ± 0.23	1.71 ± 0.42	0.160
**Muscle Regulatory Factors/Myokines**				
*PAX7*	1.00 ± 0.17	1.08 ± 0.18	0.87 ± 0.21	0.725
*MYOD1*	1.00 ± 0.12	0.92 ± 0.21	0.80 ± 0.11	0.655
*MYF6*	1.00 ± 0.04	0.99 ± 0.07	1.07 ± 0.04	0.530
*MYF5*	1.00 ± 0.07	1.29 ± 0.18	1.16 ± 0.15	0.425
*MYOG*	1.00 ± 0.07	0.84 ± 0.07	0.83 ± 0.07	0.198
*DES*	1.00 ± 0.10	1.17 ± 0.04	1.13 ± 0.12	0.473
*MSTN*	1.00 ± 0.08	1.12 ± 0.12	1.25 ± 0.14	0.368
*FST*	1.00 ± 0.10	1.14 ± 0.04	1.04 ± 0.12	0.340

All values are means ± SEM. One-way ANOVA or Kruskal-Wallis Test, and Fisher’s LSD post hoc test to determine differences among groups (different letters are statistically significantly different from each other, P ≤ 0.05).

### Fetal Pancreas Immunohistochemistry and mRNA Analysis

**Figure 7:**
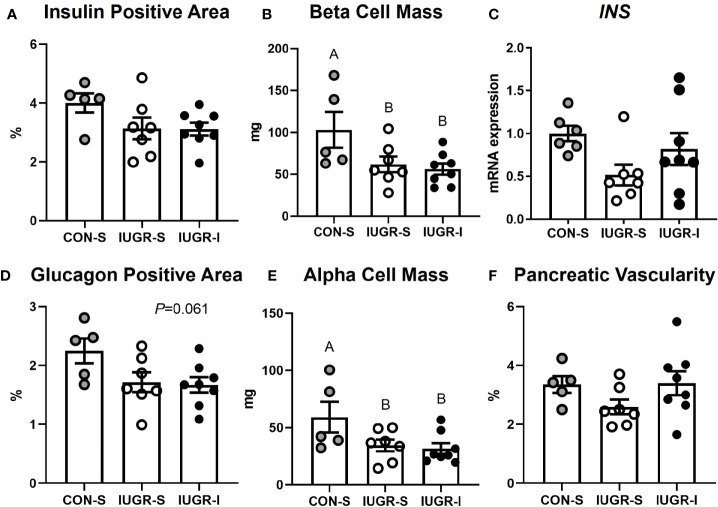
Fetal pancreas characteristics. **(A)** Insulin-positive area of the pancreas as a percentage; **(B)** β-cell mass; **(C)** mRNA expression of insulin (*INS*) shown as a fold change from CON-S; **(D)** glucagon-positive area of the pancreas as a percentage; **(E)** α-cell mass; and **(F)** percentage of pancreatic vascularity. CON-S (grey circle, n=5), IUGR-S (white circle, n=7), and IUGR-I (black circle, n=8). Values are mean ± SEM. Statistical significance was designated by different alphabetical letters at *P ≤* 0.05 by one-way ANOVA or or Kruskal-Wallis Test, and Fisher’s least significant difference *post hoc* test.

IUGR-I fetal pancreas mass tended to be lower compared to CON-S, but not different from IUGR-S (**Table 2**). There were no differences in the percentage of insulin-positive area, *INS* mRNA expression, or the percentage of pancreatic vascularity among groups. Compared to the CON-S, both IUGR-S and IUGR-I fetal pancreases had 42% and 46% less α-cell mass (*post hoc* test IUGR-I *vs*. CON-S *P*=0.020, IUGR-S *vs*. CON-S *P*=0.039), respectively, and 40% and 45% less β-cell mass (*post hoc* test IUGR-I *vs*. CON-S *P*=0.014, IUGR-S *vs*. CON-S *P*=0.032), respectively (**Figure 7**). The percentage of glucagon-positive area of the pancreas tended to be lower in IUGR-I *vs*. CON-S.

## Discussion

The main objective of this study was to test whether myoblasts within the skeletal muscle of the IUGR fetus have the potential to increase proliferation *in vivo* by direct systemic insulin supplementation at 75% gestation. We demonstrated that with a two-week insulin infusion, myoblasts from IUGR-I fetuses increased the percentage of cells in the S/G_2_/M cell cycle stages when compared to both IUGR-S and CON-S fetuses. In addition, the expression of *CCNE2*, which plays a vital role in cell cycle G_1_ to S transition, was higher in IUGR-I *vs*. IUGR-S fetuses. These insulin-promoting effects are consistent with the expected effects of exogenously supplied insulin, even though fetal plasma insulin concentrations were only minimally increased compared to baseline values. However, the total fiber number and fiber area in the FDS muscle of IUGR-I fetuses were lower compared to CON-S, and the individual fiber size and myonuclear number were not different. Also, the insulin infusion did not increase the fetal weight or organ mass in IUGR-I fetuses when compared to IUGR-S. Together, these results demonstrate that the capacity to proliferate in response to insulin remains intact *in vivo*; however, insulin alone was not able to stimulate the differentiation and fusion of myoblasts to increase myonuclear or myofiber number, or to increase fetal muscle mass. These effects occurred without feedback inhibition on β-cell development.

Previously, we reported that myoblast proliferation rates are lower by the end of an IUGR gestation compared to normally growing control fetuses and likely contribute to preferential restriction in muscle growth in the IUGR fetus (18, 21). We also demonstrated that myoblasts isolated and cultured from IUGR fetuses had a greater propensity to proliferate in response to insulin compared to myoblasts cultured from control fetuses, implicating anabolic growth factor deficiency in the IUGR fetus as a primary cause for reduced muscle mass (21). However, the myoblasts were passaged *in vitro* prior to insulin incubations and thus were exposed to a period of prolonged nutrient-enriched culture conditions, which might have contributed to correcting intrinsic deficiencies in myoblast proliferative capacity. Therefore, the current study was designed to examine the independent effect of a prolonged, low dose insulin infusion directly into IUGR fetal sheep *in vivo* at 75% gestation (112 ± 1 to 126 ± 2 dGA), when myoblasts are in the process of rapid proliferation to form new myotubes and to fuse onto existing myofibers (22, 28). Indeed, the low-dose insulin infusion shifted the IUGR-I myoblasts from the G_0_/G_1_ towards the S/G_2_/M cell cycle stages compared to both IUGR-S and CON-S myoblasts. These results are consistent with the increased proliferative response observed in normally growing fetal sheep supplemented with insulin for one-week during late gestation, as well as with intact proliferative response to insulin *in vitro* (20, 21). Therefore, our results indicate that the reduced capacity for myoblast proliferation previously observed in the IUGR fetus may be reversed by anabolic hormonal stimuli and argue against intrinsic programming of the myoblast, at least at 75% of an IUGR gestation.

However, the development of fetal skeletal muscle is not only determined by myoblast proliferation, but also by differentiation and fusion of myoblasts to form multinucleated myotubes, a process termed myogenesis. While the two-week insulin infusion into IUGR fetal sheep promoted myoblast proliferation, it did not increase the expression of myogenic regulatory factors that initiate the differentiation process, such as MyoD and myogenin, nor did it increase myonuclear number or total fiber number compared with CON-S fetuses. This was not completely unexpected, because proliferation and differentiation are considered mutually exclusive events, as withdrawal from the cell cycle is required prior to differentiation (29). Our results are consistent with previous work that showed a strong proliferative effect of insulin in postnatal ovine satellite cells with minimal effect on promoting differentiation (30). Similarly, high dose insulin along with high dose glucose in C2C12 murine myoblasts increased cell proliferation and expression of cyclins A, B1 and D and CDK4 (31). Finally, in our previous study in normally growing control fetal lambs, insulin infusion *in vivo* promoted myoblast proliferation, but it suppressed the expression of *MYF5* and *MYOG* in muscle biopsies and did not affect muscle regulatory factor mRNA expression in primary fetal sheep myotubes *in vitro* (20). However, in other studies, insulin has been shown to stimulate both proliferation and differentiation. For example, low dose insulin exposure in postnatal bovine satellite cells increased differentiation and myogenin protein expression, whereas high dose insulin stimulated proliferation and decreased the expression of differentiation factors (32). At least in fetal sheep, the predominant effect of insulin appears to be to stimulate myoblast proliferation. Alternatively, an intervention at 75% gestation may be too late, or a two-week infusion was not long enough to have significant impact on increasing myofiber numbers, considering the onset of myocyte differentiation and fiber formation is at approximately 50% gestation (85 dGA) and extends through 70-80% of gestation (22).

Insulin infusion into IUGR fetuses resulted in a rise in IGF-1 levels by day 3-4 of infusion, as has been shown previously with both acute (25, 33) and chronic fetal insulin infusions (34). IGF-1 also is a primary regulator of myogenesis. *In vitro*, IGF-1 treatment of L6A1 myoblasts results in a proliferative response during which myogenic regulatory factors are inhibited, followed by stimulation of differentiation and hypertrophy (35). Proliferation, differentiation, and stimulation of protein synthesis by IGF-1 have been demonstrated in porcine embryonic myogenic cell cultures (36, 37), cultured satellite cells from chicken biceps femoris (38), in chicken embryos (39), C2C12 murine and L6 rat myotubes (40, 41), and in primary cultured fetal myotubes (42). Similar to insulin’s effects on fetal sheep *in vivo*, we showed that a one-week IGF-1 infusion stimulated myoblast proliferation (43). Thus, it is possible that the increase in IGF-1 concentrations in IUGR-I fetuses contributed to an increase in myoblast proliferation, but did not have an effect on either myonuclear accretion or myofiber hypertrophic growth. On the other hand, mammalian target of rapamycin (mTOR) is another regulator of skeletal myocyte differentiation and myotube maturation *in vitro* with C2C12 cells (44). In C2C12 cells, mTOR has been shown to initiate differentiation *via* IGF-II in a kinase-independent signaling pathway and stimulate fusion of mature myotubes *via* kinase-dependent pathways (44). If mTOR signaling was not upregulated to a significant extent by the insulin infusion, then promotion of differentiation and fusion may not have been concurrently stimulated.

Fetuses from both IUGR groups had lower insulin concentrations compared to CON-S fetuses at baseline, as has been previously described in sheep models of placental insufficiency and human IUGR (45–47). Low-dose insulin infusion raised the IUGR-I fetal insulin concentrations compared with its baseline values beginning at day 3 of infusion and did not have a substantial influence on the IUGR-I pancreas since β-cell mass and the *INS* mRNA expression was not different from IUGR-S. Throughout the two-week infusion period, plasma insulin concentrations fell in CON-S fetuses compared with its baseline values; likely due to exponential fetal growth at this developmental period. An inverse association between gestational age and plasma insulin concentrations in cord blood at birth has been demonstrated regardless of birthweight (48). Similar trends also were observed in CON-S fetal plasma glucose, IGF-1, P_a_O_2_, and O_2_ content, where the values beginning on experimental day 9 through 14 were lower compared to baseline. A fall in plasma glucose concentrations also was observed in IUGR-S fetuses. Though we attempted to maintain euglycemia in IUGR-I fetuses with a glucose clamp, glucose concentrations were lower on days 1-14 compared to baseline. Plasma BCAA concentrations of leucine, isoleucine, and valine also were lower in IUGR-I fetuses at the end of the infusion period when compared to CON-S fetuses. Likewise, there was a fall in oxygen content in IUGR-I fetuses compared to baseline values. Myofiber hypertrophy requires a higher protein synthesis rate than protein degradation rate. Previous studies have demonstrated that low oxygen and low leucine concentrations inhibit muscle differentiation *in vitro* and *in vivo* (49–51). In a rat model of fetal growth restriction induced by maternal low protein, maternal BCAAs supplementation during pregnancy increased fetal skeletal muscle mass (52). Therefore, it is possible that subtle reductions in other energy substrates as a result of the insulin infusion limited the ability of insulin to increase muscle mass, myofiber size, or myofiber area.

At ~126 dGA, IUGR fetuses in this study were showing signs of growth restriction with decreased fetal weight, crown-rump length, pancreatic β-cell mass, and hindlimb length compared to CON-S fetuses. The skeletal muscles in IUGR fetuses including the gastrocnemius, tibialis anterior, and extensor digitorum longus weighed less than in the CON-S fetuses, and tended to weigh less when normalized to hindlimb length, indicating preferential restriction of muscle growth compared to linear growth similar to IUGR fetuses at 134 dGA as previously reported (19). In addition, at 134 dGA the FDS muscle in the IUGR fetus had fewer total myofibers, smaller fiber area, and smaller average fiber size compared to CON fetuses (18). At 126 dGA, however, the total fiber number, fiber area, and average fiber size were not significantly different in the FDS muscle between IUGR-S *vs*. CON-S fetuses. The reason may be due to the difference of 10 dGA, considering that fetal growth is exponential late in gestation and additional muscle growth in normal fetuses during this time likely will accentuate the growth lag in IUGR fetuses (53). One of the limitations of our study is the unequal fetal sex ratio in the CON-S and IUGR-S groups despite randomization. Further investigation is required to determine whether fetal sex is a significant contributing factor in the regulation of fetal myocyte proliferation and maturation. We have recently found that in the late gestation fetal sheep, with and without placental insufficiency, fetal sex does not influence placental AA transfer or blood flow (54).

In conclusion, our present study demonstrated that IUGR myoblasts have the capacity to increase proliferation *in vivo* at 75% gestation in response to a two-week insulin infusion. The direct systemic low-dose insulin infusion was enough to increase *CCNE2* gene expression and stimulate IUGR myoblasts toward the S/G_2_/M cell cycle stages without reducing pancreatic β-cell mass. Future studies are needed to determine the potential for other growth factors, such as the IGFs, that may directly promote both proliferation as well as differentiation to increase myonuclear and myofiber number. Alternatively, providing growth factors directly to the IUGR fetus at an even earlier gestational age or for a longer period may result in larger muscle mass by stimulating both myoblast proliferation and myofiber number. Encouragingly, our work has demonstrated that the IUGR myoblast is malleable and opportunities to normalize IUGR skeletal muscle development *in vivo* are an option for future therapeutic investigation.

## Data Availability Statement

The original contributions presented in the study are included in the article/supplementary material. Further inquiries can be directed to the corresponding author.

## Ethics Statement

The animal study was reviewed and approved by the University of Colorado Institutional Animal Care and Use Committee [#334].

## Author Contributions

LB conceived and designed the study. EC, BH, CM, and LB performed experiments. EC, BH, CM, PR, SW, and LB analyzed data and interpreted the results of experiments. EC prepared figures and drafted the manuscript. EC, BH, SW, CM, PR, and LB edited and revised the manuscript. All the authors approved the final version of the manuscript.

## Funding

This research was supported by the National Institutes of Health (NIH) Grants R01HD079404 (LB), S10OD023553 (LB), R01DK088139 (PR), and R01-DK108910 (SW), and by The Ludeman Family Center for Women’s Health Research at the University of Colorado School of Medicine (LB).

## Conflict of Interest

The authors declare that the research was conducted in the absence of any commercial or financial relationships that could be construed as a potential conflict of interest.

## Publisher’s Note

All claims expressed in this article are solely those of the authors and do not necessarily represent those of their affiliated organizations, or those of the publisher, the editors and the reviewers. Any product that may be evaluated in this article, or claim that may be made by its manufacturer, is not guaranteed or endorsed by the publisher.
